# Rising Canadian and falling Swedish radon gas exposure as a consequence of 20th to 21st century residential build practices

**DOI:** 10.1038/s41598-021-96928-x

**Published:** 2021-09-02

**Authors:** Selim M. Khan, Dustin D. Pearson, Tryggve Rönnqvist, Markus E. Nielsen, Joshua M. Taron, Aaron A. Goodarzi

**Affiliations:** 1 Departments of Biochemistry and Molecular Biology and Oncology, Robson DNA Science Centre, Charbonneau Cancer Institute, Cumming School of Medicine, University of Calgary, Calgary, AB Canada; 2 School of Architecture, Planning and Landscape, University of Calgary, Calgary, AB Canada; 3 Radonova Laboratories, AB, Uppsala, Sweden

**Keywords:** Lung cancer, Environmental social sciences, Natural hazards

## Abstract

Radioactive radon gas inhalation is a major cause of lung cancer worldwide and is a consequence of the built environment. The average radon level of properties built in a given period (their ‘innate radon risk’) varies over time and by region, although the underlying reasons for these differences are unclear. To investigate this, we analyzed long term radon tests and buildings from 25,489 Canadian to 38,596 Swedish residential properties constructed after 1945. While Canadian and Swedish properties built from 1970 to 1980s are comparable (96–103 Bq/m^3^), innate radon risks subsequently diverge, rising in Canada and falling in Sweden such that Canadian houses built in the 2010–2020s have 467% greater radon (131 Bq/m^3^) versus Swedish equivalents (28 Bq/m^3^). These trends are consistent across distinct building types, and regional subdivisions. The introduction of energy efficiency measures (such as heat recovery ventilation) within each nation’s build codes are independent of radon fluctuations over time. Deep learning-based models forecast that (without intervention) the average Canadian residential radon level will increase to 176 Bq/m^3^ by 2050. Provisions in the 2010 Canada Build Code have not significantly reduced innate radon risks, highlighting the urgency of novel code interventions to achieve systemic radon reduction and cancer prevention in Canada.

## Introduction

Lung cancer in people who have never smoked is now the 7th leading cause of cancer-linked death on Earth, and its prevalence is increasing^1–5^. This is driven in large part by bombardment of lung cells with alpha particle ionizing radiation through the repetitive inhalation of radioactive radon-222 (^222^Rn) gas and its decay progeny such as polonium-218 (^218^Po) and polonium-214 (^214^Po), all of which are potent alpha particle emitters^7,10–16^. Alpha particle ionizing radiation from radon damages lung cell DNA to produce genetic mutations that promote cancer, and are classified as a category 1 carcinogen by the *International Agency for Research on Cancer*^6–8^. In addition to being the principal cause of lung cancer in North American and European never-smokers, radon is also a major driver of lung cancer in smokers and causes many thousands of new diagnoses and related deaths per year^3,6,7,9–16^. Alpha particles from radon and its progeny are measured in Becquerels (Bq) per cubic meter (m^3^), equivalent to one particle emission per second per cubic metre of air. There is an additive 16% increase in relative lifetime risk of lung cancer for every 100 Bq/m^3^ of long term radon exposure^17,18^.

It is important to acknowledge that prevalent, unsafe radon exposure is a relatively recent, human-made problem rooted in the design of our built environment. Indeed, although radon is emanated by most of the Earth’s subsurface, it dilutes naturally to low levels in the atmosphere with no evident health impacts^6^. Unfortunately, the construction and design practices of the mid to late twentieth and twenty-first century have produced urban and rural environments with residential, commercial and industrial buildings that capture, contain and concentrate radon to unnatural and unsafe levels^12,13,19^. For the majority of people, radon exposure in the residential built environment is of chief concern, as it is where most of life is spent. Indeed, the typical North American will spent 68.7% of their life inside a residential building^20^. Understanding residential radon dynamics is key for projecting future exposure risks, as well as assessing the success of already implemented approaches to radon reduction, and to develop new, systematic approaches using a solid basis of performance-based outcomes.

North American residential radon exposure has worsened over time, while the opposite trend has taken place in Nordic countries^15,16,19,21–24^. Given the general similarities in climates, design trends, construction practices, technology, education, and radon awareness of both regions, it is not immediately clear why they have diverged so substantially in terms of residential radon exposure. It is important to acknowledge that there are also major differences between these regions in the prevalence of lung cancer. Primary lung cancer caused ~ 40% of Canadian cancer-related deaths in 2019, with 1 in 5 of the 29,800 new Canadian lung cancer cases in that year occurring in never-smokers^1^. By contrast, Sweden reported 4325 new lung cancers in 2019 and, adjusting for population and age profiles, this means that Canada’s annual rate of new lung cancers is currently 163% greater than that of Sweden, at 28.9 versus 17.7 new age-adjusted cases per 100,000 people per year^25^. These differences are unlikely to be explained by regional tobacco smoking rates, which are comparable at 11–13%, and have fallen in both countries with similar trajectories over recent decades^26,27^. Considering the 10–30 year latency period for lung cancer^1–5^, one plausible explanation for the disparity between Canadian and Swedish age-adjusted lung cancer incidence is that it has been driven by differences over the past several decades in exposure to other prevalent and potent lung carcinogens such as radon gas.

As 70% of the housing stock necessary to deliver on population growth projections for 2050 has yet to be built^28,29^, it is imperative to understand the etiology of evolving radon exposure trends in order to develop timely interventions to avoid a deepening public health crisis of never-smoker lung cancer in hard-hit regions. Hence, our goal was to understand the underlying factors that are (and are not) driving rising North American and falling Nordic residential radon exposure. We did this by differential analysis of Canadian and Swedish radon levels over a matching time period, and exploiting machine (deep) learning to project how radon exposure might evolve further by 2050. We also compared twentieth to twenty-first century build practices, energy efficiency provisions, radon control technology and related policies for both regions, to discern possible causative factors in diverging radon exposure trends.

## Results

### The Canadian and Swedish radon testing cohort and overall dataset trends

**Figure 1:**
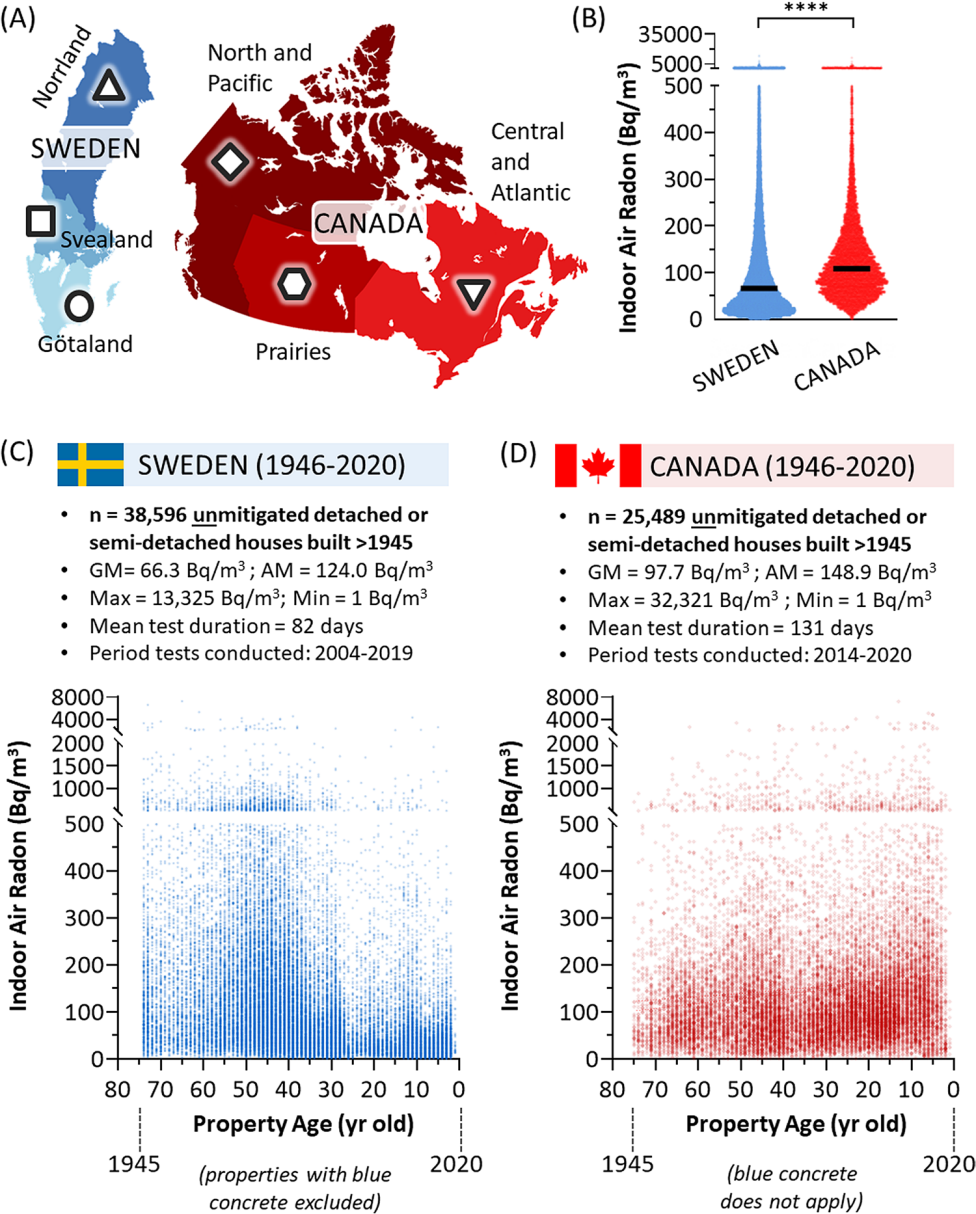
Radon test outcomes in Swedish and Canadian residential properties. Panel (**A**). Survey regions used within this study, depicting apolitical regional subdivisions of Sweden (Norrland, Svealand, Götaland) and Canada (North and Pacific, Prairies, Central and Atlantic), each of which is denoted in later figures by specific shapes (triangle, square, circle, diamond, hexagon, invert triangle, respectively). Panel (**B**). Geometric mean radon levels for the entire Canadian (red) and Swedish (blue) test cohorts. Panel (**C**). Dot plots of individual Swedish indoor air residential radon test outcomes (blue, transparency set to 50% to visualize data density) as a function of property age. Panel (**D**). Dot plots of individual Canadian indoor air residential radon test outcomes (red, transparency set to 50% to visualize data density) as a function of property age. Figures were prepared using GraphPad Prism 9.1.1 (225) (www.graphpad.com) and MapChart (www.mapchart.net).

Our survey regions included all of Canada and Sweden, which are both ‘cold climate’ countries with well established urban and rural built environments largely unscathed by recent conflict or seismic upheaval, and have comparable populations that have grown steadily over the past 75 years at broadly similar rates and population age profiles. In this study, we will use three broad clusters when considering geographic differences within each nation, shown in Fig. 1A. Our total dataset encompasses long term alpha track radon tests performed between 2004 and 2020 within households built after 1945 in Canada and Sweden. In all cases, radon test outcomes were linked to basic property metrics including year of construction, ventilation type, mitigation status, building type (and materials), and floor of testing. The same long term radon test devices were used in both regions, and captured data for an average of 82–131 days. All properties were unmitigated for radon at the time of testing and included urban and rural residential buildings of multiple types. To enable comparative analysis, multi-storey apartment buildings were not considered, as the Canadian dataset did not contain a sufficient number of properties of this type for meaningful study.

**Figure 2:**
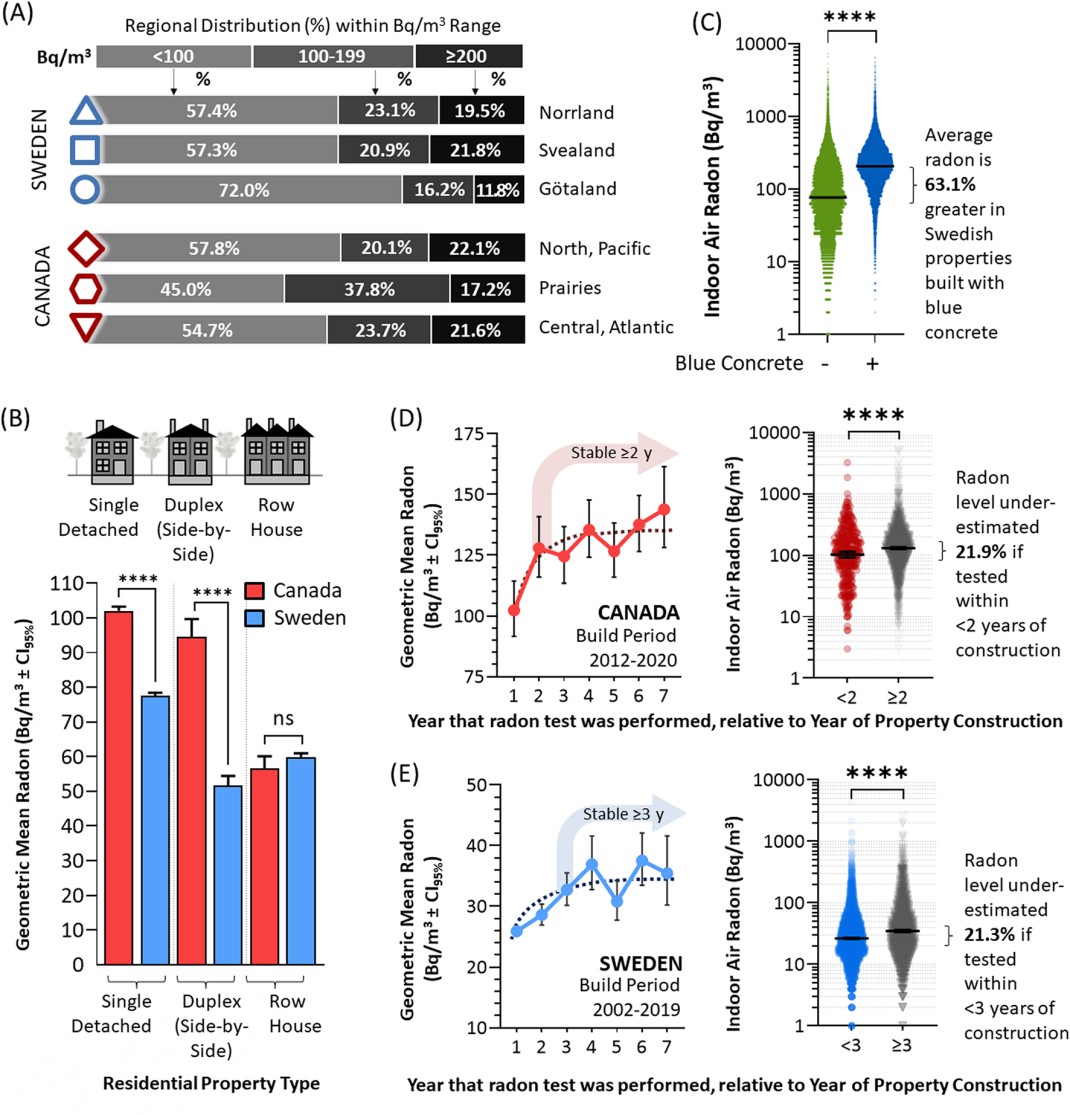
Radon outcomes by region and build type, and addressing confounding variables relating to concrete dynamics. Panel (**A**). The percentage of properties that were < 100 Bq/m^3^, 100–199 Bq/m^3^, or ≥ 200 Bq/m^3^ as by Canadian (red) and Swedish (blue) regions, as indicated and aligning with Fig. 1A. Panel (**B**). Geometric mean radon (with CI_95%_) for Canada (red) and Sweden (blue) by residential property type, as depicted in the inset cartoon. Panel (**C**). Dot plots of individual Swedish radon test outcomes in houses built using blue concrete (blue, transparency set to 50% to visualize data density) or not (green, transparency set to 50% to visualize data density). Black bars indicate geometric mean radon. Panels (**D**), (**E**). Left graphs show geometric mean radon levels ± CI_95%_ in Canada (red) and Sweden (blue) as a function of the year of testing relative to the year of property construction, with right panels showing dot plots of corresponding data (transparency set to 50% to visualize data density) grouped as indicated. Statistical analysis was done using Mann–Whitney pairwise nonparametric t-tests of dot plot data. **** = *p* < 0.0001; ns = *p* > 0.05. Figures were prepared using Excel and GraphPad Prism 9.1.1 (225) (www.graphpad.com).

Overall, the final dataset included 25,489 Canadian properties containing an arithmetic mean of 149 Bq/m^3^ radon (geometric mean 98 Bq/m^3^, CI_95%_ [96.6, 98.7], min = 1 Bq/m^3^, max = 32,321 Bq/m^3^), and 38,596 Swedish properties containing 124 Bq/m^3^ (geometric mean 66 Bq/m^3^, CI_95%_ [65.6, 67.1], min = 1 Bq/m^3^, max = 13,325 Bq/m^3^) (Fig. 1B–D). Details of the Canadian cohort have been partly described in^19^, and this is the first report of the Swedish cohort. In Canada and Sweden, 200 Bq/m^3^ is used as an administrative action level, whilst 100 Bq/m^3^ marks an exposure at or above which an increased relative lifetime risk of lung cancer is statistically significant^18,19^. To explore generalized regional risks within each country, we used the subdivisions outlined in Fig. 1A and determined the percent of properties that were < 100 Bq/m^3^, 100–199 Bq/m^3^, or ≥ 200 Bq/m^3^ as a function of geography (Fig. 2A). While there were some regional risk differences within each nation, the overall percentage of both Swedish and Canadian people experiencing excess radon risk were comparable and considered high by global standards^12^. In this study, we will also consider three distinct residential property types common to both Sweden and Canada: the single detached residence, the side-by-side (or ‘duplex’) residence, and the row house (Fig. 2B). In both nations, the single detached property contained the greatest average radon, with row-housing being lowest, and duplex (side-by-side) being variably higher in Canada or lower in Sweden.

### Minimizing confounding factors caused by the dynamics of concrete

It is important to note that we excluded Swedish properties constructed using alum shale-based aerated concrete (‘blue concrete’), a radium-containing and radon gas-emanating building material used in Swedish construction from approximately 1930 to 1980. This exclusion was because the atypical source of radon in these properties is already well-described^30^, and would confound pairwise analysis with Canada, as it is not found within Canadian buildings. We note that excluded Swedish properties containing blue concrete showed significantly (*p* < 0.0001) greater geometric mean radon (206 Bq/m^3^) versus those built without it (76 Bq/m^3^), justifying the exclusion (Fig. 2C). Radon levels may also be confounded by concrete foundation curing processes (long term drying), that continue for 1–2 years, and can expose wall-to-foundational gaps and cracks that enable greater radon entry^31–33^. To examine this, we grouped all properties that were both built and tested between 2004 and 2020, and compared radon levels measured in the same (< 1) or subsequent years relative to building completion and owner occupancy (Fig. 2D, E). In both Canada and Sweden, radon levels measured in the first year that a property existed were significantly (*p* < 0.0001) lower (~ 21%) versus those obtained in later years. This premature radon testing underestimation effect was comparable between Sweden and Canada, although radon readings stabilized ≥ 2 years post construction in Canada, and ≥ 3 years in Sweden. To avoid ‘false low’ readings from confounding outcomes when considering data as a function of build year, we excluded these prematurely-conducted radon tests from all further analyses, including Fig. 2A, B.

### Time series analysis of Canadian and Swedish radon as a function of construction period

**Figure 3:**
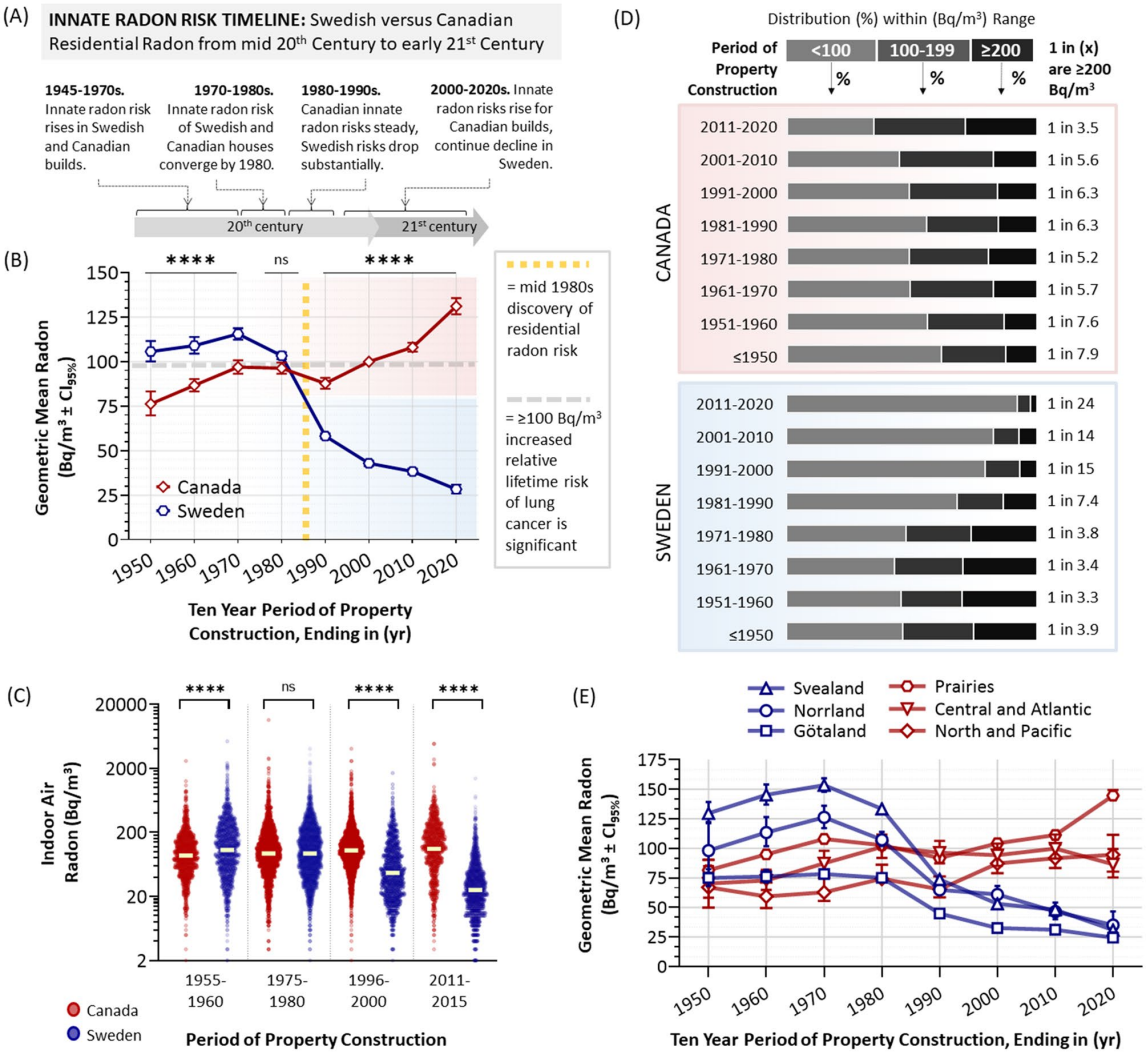
Time series of innate radon risk in Swedish and Canadian properties as a function of period of construction. Panel (**A**). An annotated timeline of major fluctuations in innate radon risk of a property from the twentieth to the twenty-first century. Panel (**B**). Geometric means ± CI_95%_ for 10-year periods of residential property construction (x-axis shows year ending decade, e.g., 2020 corresponds to 2011–2020, 1990 corresponds to 1981–1990, etc.) for Sweden (blue line with hexagons) and Canada (red line with diamonds). The vertical yellow dotted line indicates the period (1984) that the risk of high radon in residences was first documented globally. The horizontal grey dashed line indicates the radon level (100 Bq/m^3^) at or above which a statistically significant increase in relative lifetime risk of lung cancer is evident. Panel (**C**). Dot plots of source data from (**B**), clustered into sample 5-year periods for Canada (red) versus Sweden (blue), with transparency set to 50% to visualize data density. Panel (**D**). The percentage of properties that were < 100 Bq/m^3^, 100–199 Bq/m^3^, or ≥ 200 Bq/m^3^ as by Canadian (red) and Swedish (blue) periods of property construction (as indicated). Panel (**E**). The same data as in (**B**), but split into the Canadian (red) and Swedish (blue) regions, as indicated and aligning with Fig. 1A. Statistical analysis was done using Mann–Whitney pairwise nonparametric t-tests of dot plot data or 1-way ANOVA for all other data. **** = *p* < 0.0001; ns = *p* > 0.05. Figures were prepared using Excel and GraphPad Prism 9.1.1 (225) (www.graphpad.com).

**Figure 4:**
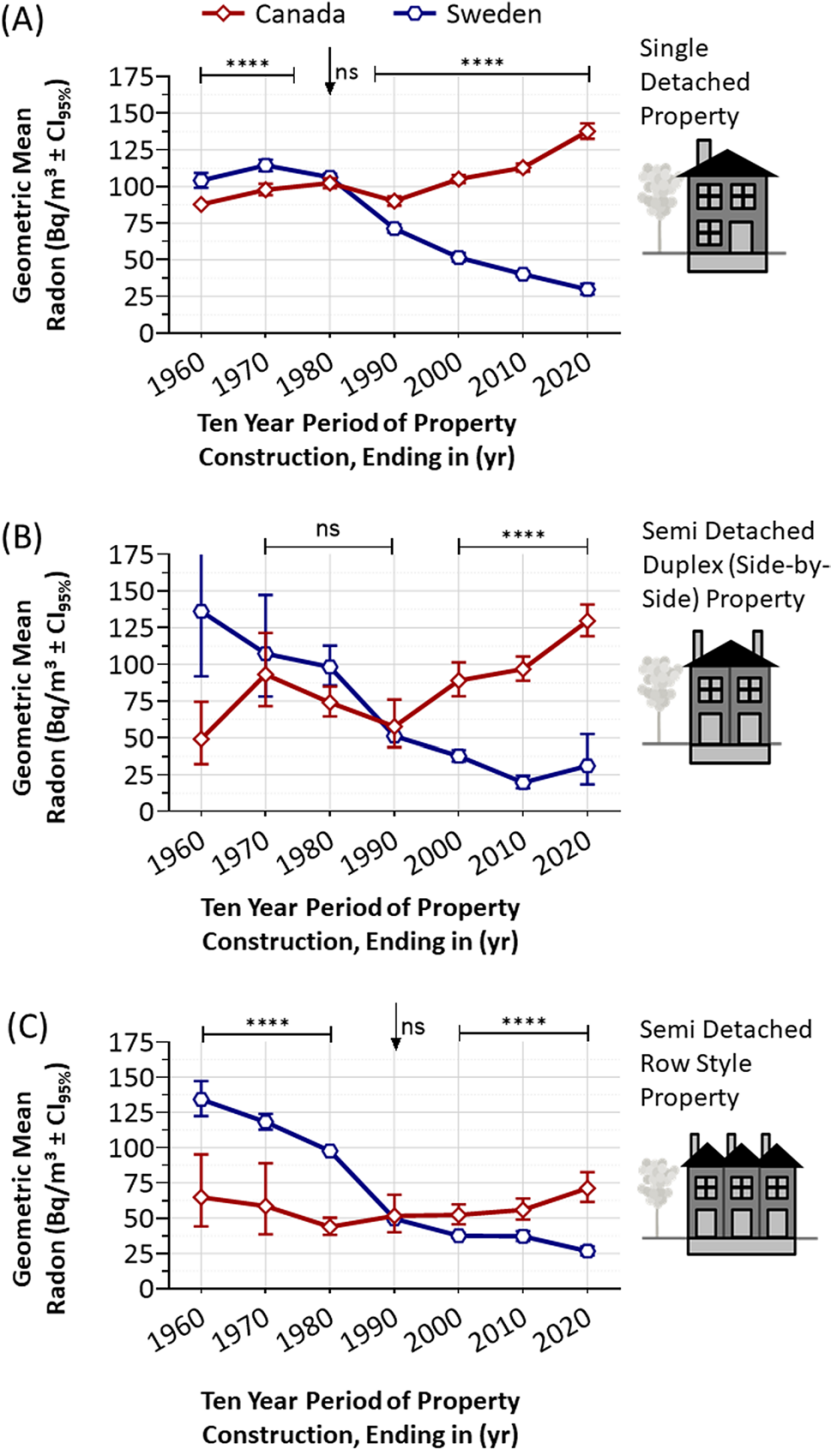
Time series of innate radon risk in different types of Swedish and Canadian properties, as a function of period of construction. Geometric means ± CI_95%_ for 10-year periods of residential property construction for Sweden (blue line with hexagons) and Canada (red line with diamonds). Panel (**A**) includes all single family detached properties, as depicted in the inset cartoon. Panel (**B**) includes all semi-detached side-by-side (also called duplex, triplex or quadruplex) properties, as depicted in the inset cartoon. Panel (**C**) includes all semi-detached row-style properties, as depicted in the inset cartoon. Statistical comparisons are pairwise t-tests for Canada versus Swedish data for a specific decade of construction. **** = *p* < 0.0001; ns = *p* > 0.05. Figures were prepared using GraphPad Prism 9.1.1 (225) (www.graphpad.com).

There are clear differences in the data distribution of Swedish and Canadian residential radon levels as a function of property age (Fig. 1C, D; Table 1). To better measure these trends, we clustered test outcomes into 10-year groupings by year of property construction. We then calculated the geometric mean radon observed within residences built in each period and considered this value to reflect the changing ‘innate radon risk’ within the built environment of each region (Fig. 3A–C, Table 1). The trends demonstrated a striking convergence and divergence of residential radon exposure in Canada and Sweden. Residential radon levels are consistently and significantly (*p* < 0.0001) greater in Swedish versus Canadian residences built 1951–1970. For properties built in the 1970s, however, radon levels between each nation converge, are comparable (96 Bq/m^3^ in Canada, 103 Bq/m^3^ in Sweden) (Fig. 3A, B), and were not statistically (*p* > 0.05) different (Fig. 3C). After 1980, however, innate radon risk trends between these regions diverged by a large margin and by the 2011–2020 period had risen in new Canadian builds to 131 Bq/m^3^, while decreasing steadily in Sweden to 28 Bq/m^3^—a now modern difference of 467% between each country. This trend is also evident by analysing the percentage of properties that were < 100 Bq/m^3^, 100–199 Bq/m^3^, or ≥ 200 Bq/m^3^ as a function of decade of property construction, whereby a contemporary new property in Sweden has a 1 in 24 chance of exceeding 200 Bq/m^3^, while the Canadian equivalents have a 1 in 3.5 chance (Fig. 3D). The decline in innate radon risk in Swedish properties occurred in an equivalent manner across all Swedish regions examined, with Svealand and Norrland experiencing the largest relative decrease (Fig. 3E). In Canada, all regions also experienced a rise in radon over most of the twentieth to twenty-first century, with this being proportionately largest in the Prairie region. We note that residential indoor air radon levels in Atlantic and Central region of Canada, although not significantly decreasing from 2001 to 2020, also did not experience the most recent rises in innate radon risk that occurred in the remainder of Canada. The reasons for this are not clear and warrant future investigation. However, as these trends are consistent across different property types in both Sweden and Canada (Fig. 4), we suggest that the etiology of regional trend differences are not directly related to any gross disparities in property type distribution.

**Table 1:** Summary of radon in Swedish and Canadian properties as a function of period of construction.

Build period	n (# datapoints)		Geometric mean radon (Bq/m^3^)		Upper CI95		Lower CI95	
	Canada	Sweden	Canada	Sweden	Canada	Sweden	Canada	Sweden
1941–1950	386	1385	76.3	105.7	83.3	111.6	69.8	100.1
1951–1960	1679	2204	86.7	109.0	90.3	113.8	83.3	104.5
1961–1970	1965	4945	97.0	115.6	100.8	118.8	93.2	112.4
1971–1980	3162	10,190	96.3	103.3	99.5	105.3	93.3	101.3
1981–1990	2562	7262	87.8	58.2	90.9	59.7	84.8	56.7
1991–2000	3221	3459	100.0	42.9	102.8	44.4	97.2	41.4
2001–2011	4307	2847	108.1	38.3	110.6	39.9	105.6	36.8
2011–2020	2392	617	131.1	28.4	135.7	30.9	126.6	26.1

### Swedish and Canadian radon in relation to build code and energy efficiency policies over time

**Figure 5:**
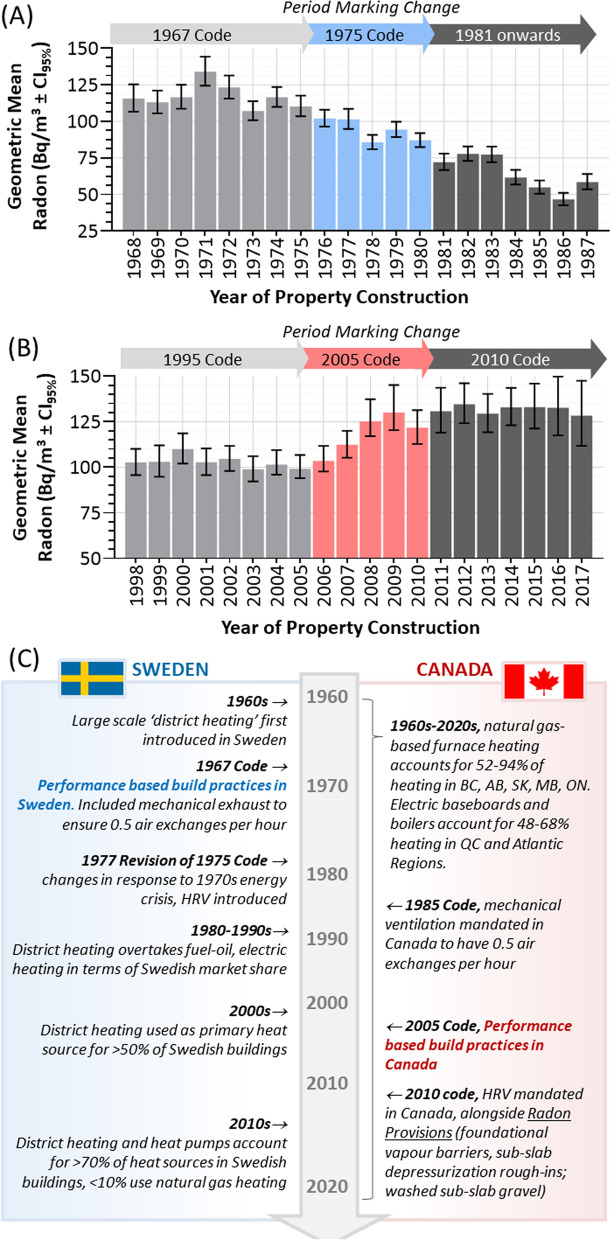
Periods marking notable changes in Swedish and Canadian residential property innate radon risk, and key build code and energy use trends since 1960. Panel (**A**). Yearly geometric mean radon levels ± CI_95%_ for residential property construction in Sweden from 1968 to 1987, indicating build codes that applied during each year. Panel (**B**). Yearly geometric mean radon levels ± CI_95%_ for residential property construction in Canada from 1998 to 2017, indicating build codes that applied during each year. Panel (**C**). Timeline schematic of 1960–2020, indicating relevant events in Sweden (left, blue) and Canada (right, red). Figures were prepared using GraphPad Prism 9.1.1 (225) (www.graphpad.com).

While the innate radon risks of a property built in Canada and Sweden in 1980 were essentially the same, the data outlined in Figs. 1, 2, 3, and 4 present a striking case of very different outcomes over time. It is reasonable to hypothesize that subsequent changes in design trends and/or build codes over the following 40 years (1981–2020) underlie the significant increase in Canadian radon, and the opposing situation in Sweden. It is important to note that Canadian national building codes have no legal status until they are accepted by the provincial legislatures and municipal government bylaws^34^, a process that can take up to 5 years^35,36^. This means that realized changes in Canadian build practice are typically spread out in time. In contrast, Swedish national building codes are mandated from their publication, and result in more immediate changes in practice^37–40^. With this in mind, we note two different 20-year periods in each nation of rapid innate radon risk change that warrant closer examination. These are 1968–1987 in Sweden, where innate radon risks began to progressively fall in new properties (Fig. 5A), and 1998–2017 in Canada, where risks suddenly began to increase (Fig. 5B). In both cases, these periods coincided with the introduction of performance-based objective build code practices, as well as a variety of energy efficiency provisions^41–43^ (Fig. 5C).

Performance-based design and construction requires an entire building to meet measurable requirements, such as energy efficiency, air ventilation, or seismic load, and contrasts with prescriptive-based building practices that require a builder to satisfy specified (often numeric) standards for individual items, such as a given R-value in roof insulation^37,40^. Sweden introduced performance-based objective building code regulations in 1967, while the same style of build code was only published in Canada in 2005 and took up to 2010 to be fully adopted. Based on our observation that innate radon risks in Canada and Sweden diverged with opposite trajectories as performance-based build code practices were adopted, we suggest that the adoption of this design and building philosophy is, itself, NOT directly correlative or causative with higher or lower radon in the built environment.

### Swedish and Canadian radon as a function of energy efficiency-related ventilation changes

We next examined more identifiable changes occurring in each nation’s build code during the periods of change marked in Fig. 5. In both Sweden and Canada, new functional requirements relating to residential energy efficiency coincided with the introduction of performance-based build practices. These changes intentionally produced more air-tight properties and, in turn, necessitated more sophisticated controls over building ventilation to ensure a healthy balance between fresh and stale air.

**Figure 6:**
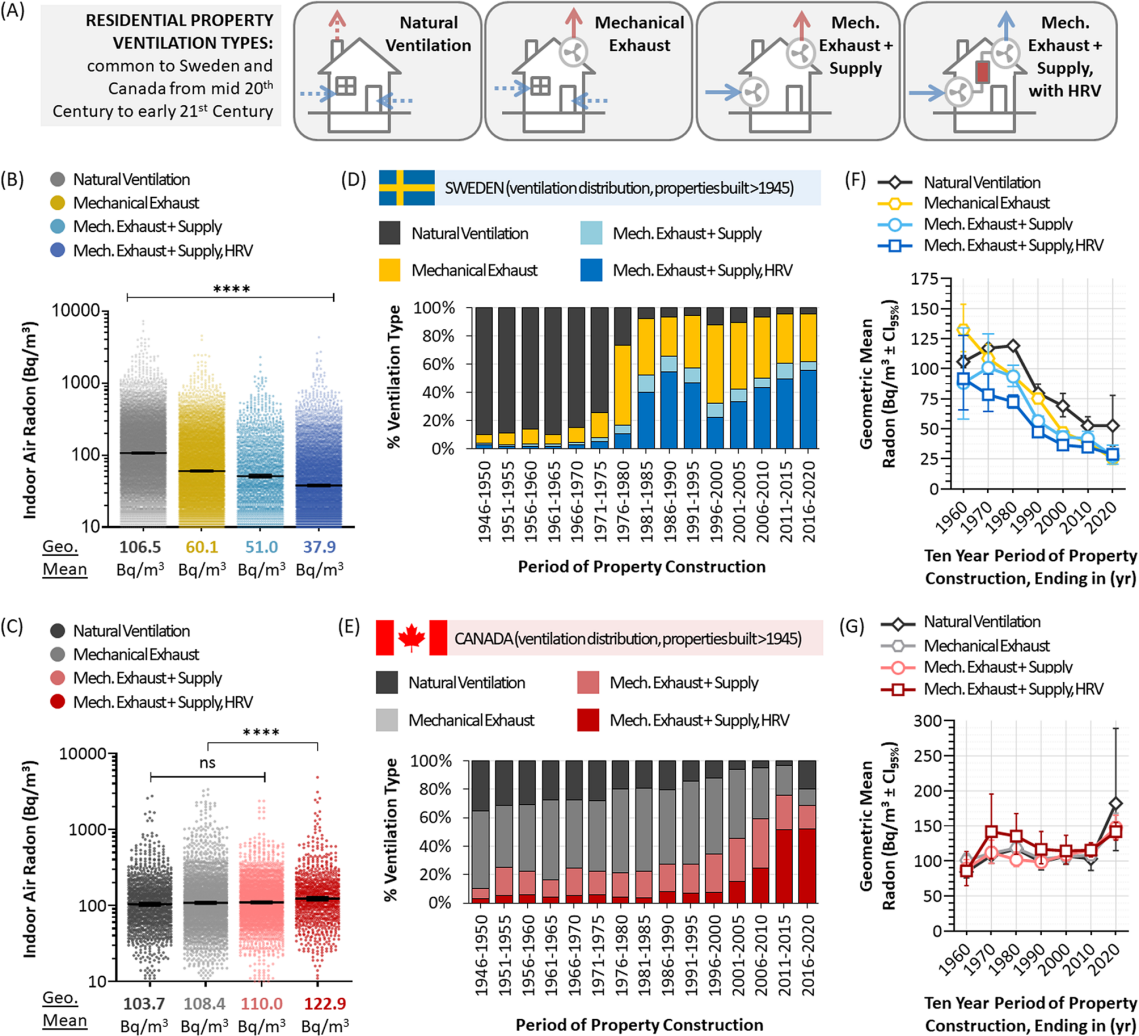
Innate radon risks in Swedish and Canadian residential properties as a function of ventilation type. Panel (**A**). A depiction of the four major types of residential building ventilation in Sweden and Canada. Panels (**B**), (**C**). Dot plots of radon in Swedish (**B**) and Canadian (**C**) properties as a function of ventilation types from (**A**), with transparency set to 50% to visualize data density. Geometric mean radon values are shown below the graph. Panels (**D**), (**E**). The percent distribution of each ventilation type from (**A**) over time in Sweden (**D**) and Canada (**E**). Panels (**F**), (**G**). The geometric means ± CI_95%_ for 10-year periods of residential property construction (x-axis shows year ending decade, e.g., 2020 corresponds to 2011–2020, 1990 corresponds to 1981–1990, etc.) for Sweden (**F**) and Canada (**G**). Statistical comparisons are Mann–Whitney pairwise nonparametric t-tests of comparisons for dot plot data or 1-way ANOVA for all other data. **** = *p* < 0.0001; ns = *p* > 0.05. Figures were prepared using Excel and GraphPad Prism 9.1.1 (225) (www.graphpad.com).

To determine how shifts in property ventilation impacted radon, we analyzed innate radon risks over time as a function of four ventilation types: (1) natural ventilation, (2) mechanical exhaust, (3) mechanical exhaust and supply, and (4) mechanical exhaust and supply with heat recovery ventilation (HRV) technology (Fig. 6A). In Sweden, there were significant differences (*p* < 0.0001) in radon levels between all four ventilation types, with properties relying on natural ventilation being highest for radon, and those with mechanical supply and exhaust with HRV being lowest (Fig. 6B). In Canada, however, properties ventilated by mechanical supply and exhaust with HRV had the highest amount of radon relative to the other three (Fig. 6C). Unlike Sweden, there were no statistically significant (*p* > 0.05) differences between natural ventilation and those with mechanical exhaust and/or supply. To examine this more closely, we monitored the relative prevalence of each ventilation type over time in Canada and Sweden (Fig. 6D, E), and found that they reflected the known timeline of adoption within each nations build code (Fig. 5C), with HRV-based ventilation rising in prominence in Sweden after 1980 and in Canada only after 2010. On the surface, HRV adoption in Sweden during the 1980s correlated with the most substantial period of reduced radon risk in that nation, while the opposite is true in Canada during the 2010s. By examining innate radon risk across all four ventilation types as a function of construction period (Fig. 6F, G), it became clear that adoption of these ventilation types was independent of radon. Indeed, the innate radon risk of properties with all four types of ventilation changed together with comparable patterns, either all rising (Canada) or falling (Sweden) in relative synchrony. We conclude from this that the adoption of heat recovery ventilation, in of itself, is also NOT a fundamental driver of radon risk.

### Impact of radon reduction provisions in the 2010 Canada build code

**Figure 7:**
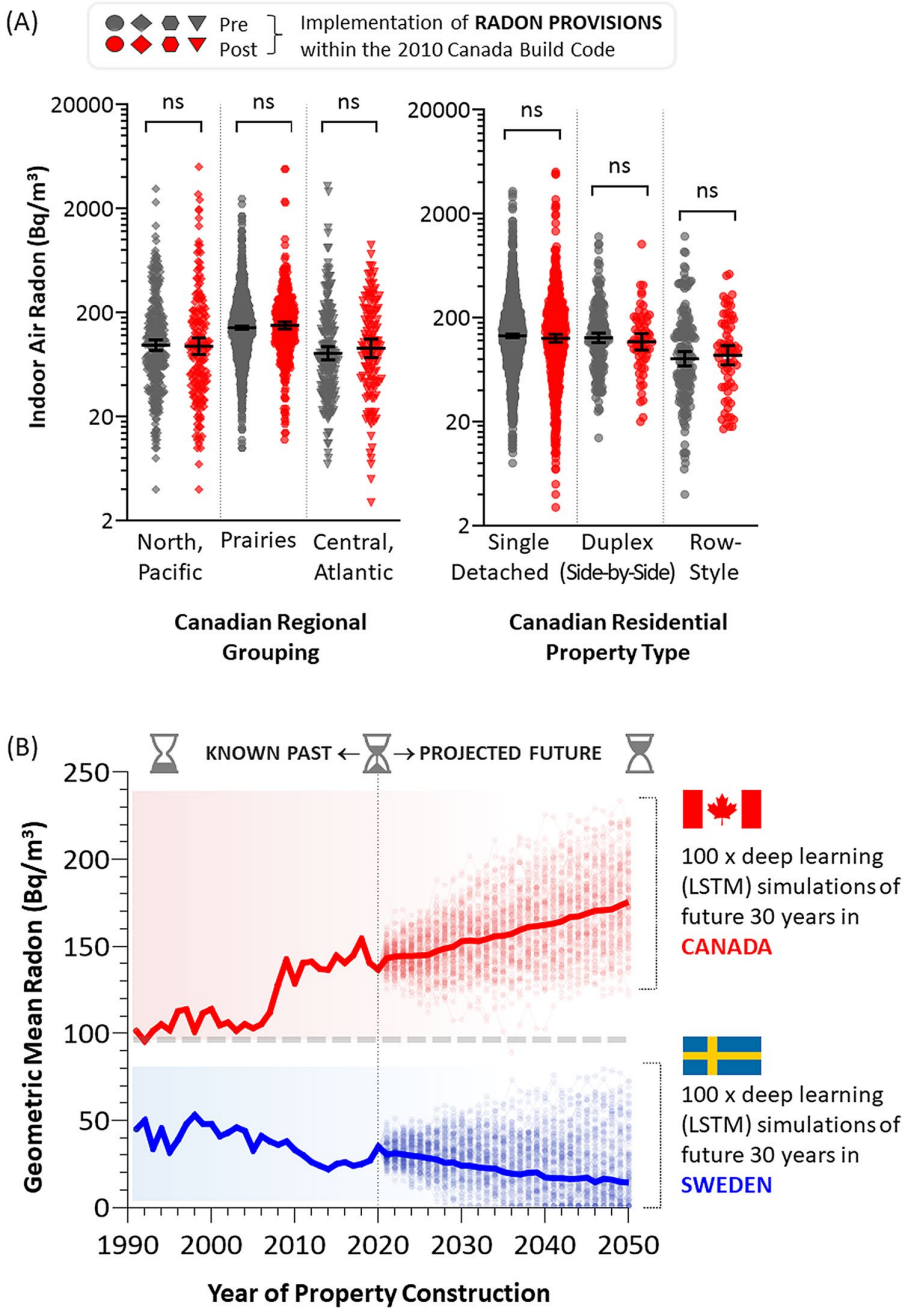
Impact of the 2010 Canada build code on radon, and deep learning predictions of radon in Canada and Sweden by 2050. Panel (**A**). Dot plots of radon as a function of region (left) and property type (right) in Canadian properties built up to 10 years before (grey symbols) the 2010 Canada Build Code was adopted or after (red symbols). Statistical comparisons are Mann–Whitney pairwise nonparametric t-tests ns = *p* > 0.05. Panel (**B**). Deep learning predictions of future innate residential radon levels between 2021 and 2050, as a function of known trends from 1991 to 2020. Solid lines (red for Canada, blue for Sweden) represent known geometric mean radon for the period before 2021, or the geometric average of 250 long short-term memory (LSTM) network predictions (shown as 50% transparent lines with dots) for the projected future up to 2050. Figures were prepared using GraphPad Prism 9.1.1 (225) (www.graphpad.com).

To date, and to our knowledge, Sweden has not introduced any specific build code change that explicitly incorporates radon reduction provisions; however, it certainly has detailed ventilation codes, radon testing guidelines, and provisions regarding blue concrete^40^. Given the already systemic reductions in Swedish radon illustrated by our data here, this is not an issue. By contrast, Canada’s radon problem remains high, and is still growing—meaning that future systemic changes are needed to reverse current trends. In 2010, Canada introduced several measures to its build code that aimed to improve radon reduction^35^. These included (1) a sub-(concrete) slab depressurization ‘rough-in’ to all building foundations, (2) increased washing required of sub-foundation gravel layers (to eliminate fine particulate that reduces gas communication below the slab), and (3) the inclusion of a plastic vapour barrier between the gravel and concrete foundations. To determine whether these measures had any impact, we monitored radon levels in Canadian properties build after the 2010 code was adopted^34–36^ and compared this to radon levels in properties built in the preceding (up to) 10-year period. We note that Canadian provinces variably adopted the 2010 code between 2011 and 2015, and so the cut off we used for each before and after (code adoption) period was set in a regionally specific manner for greatest sensitivity. This analysis was then performed as a function of Canadian region (as in Fig. 1A) and also property type (as in Fig. 2B). We found no statistically significant (*p* > 0.05) effect on radon, with all properties constructed after adoption of the 2010 Canada build code containing the same overall innate radon risk as those build during most immediate previous period (Fig. 7A). This was not entirely surprising, given that the radon-related provisions introduced to the 2010 code would not, in theory, suppress radon entry but rather were intended to make it easier to mitigate for radon at a future date. This indicates that novel, more impactful changes to future Canadian build codes are still needed to reduce innate radon risk in new residential properties.

### Deep-learning prediction of future residential property innate radon risks to 2050

Finally, we used deep learning to model how the innate radon risk of residential properties in Canada and Sweden might evolve over the next 30 years. Deep learning is a type of machine learning based on artificial neural networks, in which multiple layers of processing are able to progressively extract higher level features from large, often complex datasets. We previously developed a deep learning method applicable to understanding radon in the built environment^44^, and applied a refinement of that initial technique to the datasets in this study. Briefly, this involved a ‘long short-term memory’ (LSTM) network, a type of artificial, recurrent neural network with feedback connections capable of learning order dependence in sequence prediction problems (see methods for details). We used all available property metrics from the 30-year period previous to 2020, and used those to make predictions for the next 30 years (to 2050). The model assumed that no radon-specific changes to future build codes (beyond what had already occurred, and were trending over the previous 30 year period) took place. This was by design, in order to illustrate what the consequences of inaction might be. Each model was run 250 times and then aggregated together to develop a consolidated prediction of innate radon risk per year (Fig. 7B). The models predict that Swedish innate radon risk may continue a modest decline to background levels (< 15 Bq/m^3^), while Canadian levels might rise such that a residential property built in 2050 would contain an average of 175 Bq/m^3^.

## Discussion

Understanding innate radon risks—the Bq/m^3^ geometric mean radon level of a property constructed in a given period—is an important step in identifying key points of action to reduce cancer-causing levels of radon inhalation in the residential built environment. This work finds that Sweden has successfully done this, and that radon has been functionally ‘engineered out’ of new Swedish houses (all types, all regions), albeit ‘unintentionally’, insofar that no explicitly radon-related measure has been introduced to their build codes to date. In striking contrast, Canada’s residential radon exposure problem has persisted through the twentieth century and increased even further in the twenty-first century. Our modelling predicts an exacerbation of this over the next 30 years and, hence, reveals a troubling Canadian future in lung-cancer causing particle radiation exposure unless meaningful radon-reduction interventions are implemented with urgency. We emphasize that younger populations with children in North America are more likely to reside in newer properties with the largest doses and dose rates of radiation from radon (to the lungs)^19^, making the need to address this issue even more tangible as these demographics of people are also more prone to negative health effects from radon^13–15,45–48^. We assert that engineering radon exposure ‘out’ of the built environment is one of the most impactful strategies that can be employed to prevent lung cancers. A majority of global radon prevention strategies aim to convince individuals to test and personally invest in post-construction radon mitigation solutions if a building exceeds regional action thresholds^47,49^. The overall efficacy of this strategy is limited in scope, as it relies on a large number of human psychological, sociological, economic, and behavioural variables to persuade individuals to perform a radon test, understand the outcomes, and effectively mitigate properties—a process that is neither inclusive nor equitable at present^50^. By contrast, wholesale systemic approaches to removing radon from entire inventories of properties (e.g., anything built after a certain date), are likely to be faster, more equitable, and certainly more impactful from a population-based cancer prevention perspective.

The causes underlying opposing trends between Canadian and Swedish innate residential radon risks are complex, with no single, ‘obvious’ event or build code change that either reduced or increased radon in either country. Our data argues against the adoption of performative build code philosophies or heat recovery ventilation technology as directly influencing high versus low radon, as the exact opposite outcomes were obtained in Canada and Sweden when each was introduced. However, it is possible that, within the specific context of each region’s built environment, that a single change (such as including HRV units) might have opposing effects. We note that there is a major difference between Canada and Sweden in terms of how properties are heated, with natural gas-based furnaces (57%), electric baseboard heaters (27%), and boilers (radiators) (5%) encompassing the vast majority of heating in Canada^51^ (Fig. 5C). By contrast, Sweden began to phase these methods out during the mid twentieth century, replacing them with district heating^52^. District heating uses the combustion of biomass fuels in a centralized facility to produce steam that is then forced through a pipe network to individual properties for radiant heat distribution^52^. By the 2010s, district heating accounted for > 70% of heating in Sweden, while natural gas-based furnaces encompassed < 10%. Natural gas-based furnaces require forced-air ventilation from lower to upper property levels to distribute heat, a process that has major implications to air dynamics and pressures within a given building. We raise the possibility that the prevalence of this heating type in Canada (especially the Prairie region, where it accounts for 77–94% of all heating types) might be a major reason why introducing HRV has corresponded with an increase in innate radon risks, versus the decreases observed in Sweden and elsewhere^53^. Indeed, HRV units have the potential to reduce radon by 25–75% in some residential buildings^53^; however, this effect is often precluded or even reversed when ‘complicated’ by a forced air ventilation system where fresh air intake and stale air efflux balances may be compromised. Future work will be required to specifically measure, model, and verify whether this is the case, especially to model the complexities of building ventilation rates on radon over time, which is a synthesis of not just of heating and ventilation system, but also the presence of air conditioning, chimneys, roof insulation, window glazing and age, human behaviour, season and more. However, irrespective of this, it is not reasonable to expect a rapid, universal change in heating type in new Canadian properties over the near term, nor can the vast, existing built environment be converted wholesale with any degree of speed or economy.

So, what can be done about radon in Canada with urgency? We suggest that one immediate and potentially cost-effective solutions to this issue—also proven as effective—is the inclusion of a complete sub-slab depressurization (radon mitigation) system in all new builds. If installed at construction, costs are transferred from property owners to builders, but are counterbalanced by the economy of scale that make systemic radon reduction far more economical versus ad hoc retrofits to already completed buildings. A complete economic cost–benefit analysis of this is warranted. The next Canadian build code is due to be published in 2025, and so there is a near-term opportunity to introduce such measures that would be expected to take effect across the nation by 2030. We want to emphasize, however, that the existing built environment constitutes the theater within which a radon exposure problem currently exists in Canada and Sweden, and constitutes the majority of the built environment itself. Hence, retrofit solutions are and will remain absolutely necessary if rapid changes to population health are a targeted objective. A detailed analysis of all property metrics contributing to high radon in Canada is also still important, as this can be used to develop predictive models for radon awareness programs and a demographically sensitive series of public health interventions. However, we assert that ‘waiting’ for these outcomes (from a build code change perspective) is not logical due to the scale of contemporary Canadian radon exposure, as it is likely that many building features that exacerbate radon exposure may not be possible to alter while also meeting energy efficiency needs. It is clear from the Swedish example that greater energy efficiency can be achieved whilst also reducing radon—and so, although these two things are related, they are not fundamentally linked together.

In summary, we find that North American and Scandinavian residential property innate radon risks have changed substantially over time, such that new Canadian houses are being constructed with 467% greater radon versus their Swedish equivalents. In terms of possible limitations to our work, we acknowledge that our sampling methods in Canada have had some bias toward the Prairie region, as this is where recruitment commenced. However, by performing a regionally sensitive analysis, we have ameliorated this as much as possible. We also acknowledge that in both Sweden and Canada we have excluded multi-family dwellings such as high rise apartment buildings. Further data will be required to determine whether our observations here also apply within that residential environment, as well as occupational environments. Aside from the known issue in Sweden relating to ‘blue concrete’, radon risks cannot yet be predicted accurately by property type, and so we do not consider it likely that our data contains biases towards low or high radon-containing properties based on a willingness of radon-testing occupants to participate. We stress that there is no blame (or credit) that can be attached in any group or persons for rising Canadian and falling Swedish innate radon risks, although meaningful future intervention to reduce high Canadian radon exposure should be addressed as fast as achievable by all those in a position to do so. Until then, radon in residential properties will continue to drive the formation of radon-induced lung cancers in this region, at a substantial cost in human suffering.

## Methods

### Participant eligibility and enrollment

All radon-testing and research activities in Canada (including data agreements with teams in Sweden) were pre-approved by the Conjoint Health Research Ethics Board, Research Services, University of Calgary (IDs = REB17-2239, REB19-1522), adhering citizen science research best practice^54^. All methods (here and below) were carried out in accordance with national and local guidelines. Records of informed consent were obtained in all cases. Participants were permitted to withdraw at any time. As part of this work, people living in Canada and Sweden purchased alpha track 90 + day radon detectors that they then deployed, returned for analysis, and later received their specific radon reading in a confidential manner. Radon testing in Canada and Sweden was based on random recruitment for all wanting to test and/or join citizen-science based radon testing projects, with all adult homeowners and renters in any residential building type being equally eligible. Prior to radon testing, it is very difficult for any resident within a given region to accurately predict that their property may have ‘high’ or ‘low’ radon, reducing this potential selection biases to the fullest extent. No data from any constituent part of this cohort were from known or pre-selected lung cancer cases. Commercial offices or hospitality service buildings were not considered.

### Radon testing and surveying

All radon tests are closed passive etched track detectors made from CR-39 plastic film inside antistatic and electrically conductive housing with filtered openings to permit gas diffusion, with a typical linear range of < 15–25,000 Bq/m^3^ for a 90 day reading. Calculation of values falling outside of normal linear ranges were possible for those tests conducted for longer periods, where alpha hits on CR-39 chips were clearly and unambiguously delineated via microscopy and quantitation software. To be read, CR-39 films are etched in 5.5 N NaOH at 70 °C for 15.5 min and scored using TrackEtch® software at ISO17025 accredited laboratories (Radonova Laboratories, Sweden). Participants were instructed to place tests on the lowest level of the property that a person spends ≥ 4 h per day (this is based on national radon test guidelines). Complete details of calibration controls, including duplicates, blanks, and spiked positive controls used to establish precision and accuracy for this study protocol have been described in detail in^14^. In brief, 5% of all tests are randomly selected for a duplicate test, placed < 10 cm from the primary device. The r^2^ value for duplicates is > 0.96. In Canada, non-profit ‘Evict Radon’ national study kits were available to participants for between CAD$ 45 and 52 (Canadian dollars) with cost depending on study year and differences driven by inflation over the period. In Sweden, the identical radon test devices cost between SEK 250 and 600 (Swedish krona), with costs also rising with inflation over the period. Participants all completed online basic property metric surveys at the time of radon kit registration, and these surveys have been described before^14^.

### Deep learning analysis

Descriptive and time-series analyses were conducted using both traditional Auto-Regressive Integrated Moving Average (ARIMA) and newest deep learning time series forecasting toolsets in MATLAB2020b using codes as well as the TSFA econometric platform. Descriptive statistics of the concentrations of radon levels and year of construction were analyzed, filtered, random fluctuations were identified, and then appropriate models were trained to forecast and compare radon levels in houses build from 1945 to 2020 in Canada and Sweden. As for the time series prediction, historic data should be stationary where the covariance of the variable of importance is a function of lag, not of time. We found both Canadian and Swedish datasets were non-stationary through Adfuller tests (i.e., they both had trends and seasonality), and so we removed these through differential filtering and decomposition to get the stationary data with random fluctuations of radon levels suitable to assign to an ARIMA model. We used Fourier Transformation (that provides spikes in the frequency domain corresponding to the number of harmonics) to multiply the signal to remove seasonality. We used autocorrelation (AC) and partial autocorrelation (PAC) to determine autoregression as follows: (1) if AC tailed off gradually and PAC cut off after p lags = AR(p) model; (2) if AC cuts off after q lags, and PAC tails off gradually = MA(q) model; (3) if both AC and PAC tails off gradually = ARMA (p. q) model. In our data, AC and PAC tailed off gradually, so we integrated both AR(p) and MA(q) models into ARIMA model. Our loaded case data contained a time series where the time steps corresponded to year of construction and values corresponded to the radon test results. The output was a cell array, where each element was a single time step. We trained the first 90% of the sequence and tested on the last 10% for sequence-to-sequence regression network. The LSTM network architecture had 1 input and 1 output variable, 200 hidden neuronal layers; we ran 100–250 epochs, setting gradient threshold at 1 and piecewise initial learning rate at 0.005 with a 20% drop factor from the mid point (numFeatures = 1; numResponses = 1; numHiddenUnits = 200; ‘MaxEpochs’, 250, ‘GradientThreshold’, 1, ‘InitialLearnRate’, 0.005, ‘LearnRateSchedule’, ‘piecewise’, ‘LearnRateDropPeriod’, 125, ‘LearnRateDropFactor’,0.2). The training progress plot reported the root-mean-square error (RMSE) calculated from the standardized data. Once the model was trained, we tested it to predict forecasted values and compared that with the test data. To forecast the values of future time steps of a sequence, our trained LSTM model produced responses with the sequenced values shifted by one time step. Where, at each time step of the input sequence, the model learned to predict the value of the next time step. To forecast the values of multiple time steps in the future, we used the predictAndUpdateState function to predict time steps one at a time and update the network state at each prediction. We applied the model to display 1991–2020 and projected future 2021–2050 radon levels. This model performed better in dealing with large volume of data and produced more accurate outcomes in terms of prediction errors (as measured with RMSE) that is the standard deviation of the residuals showing how close the data points are from the best fit regression line compared to that in the traditional ARIMA model which has some other limitations.

### Statistical analysis

Statistical analysis was carried out using Excel, Prism and R (4.0.2). One-way ANOVAs were carried out to test differences between groups (e.g., year of construction, occupant age, mSv, etc.), with Bonferroni–Holm post-hoc testing carried out to characterize group differences for pairwise comparisons if the ANOVA reached significance.

## Data Availability

The de-identified raw data sets generated by the current study are available to academic researchers at public institutions following reasonable request to the corresponding author of this study, and will require a data transfer agreement. Data may not be used for private, commercial, or for-profit purposes for any reason. Complete coding details for all Deep Learning protocols are also available on request.
